# Mental health and addiction in young refugees-Research on prevalence of alcohol and substance use, PTSD and psychological difficulties experienced by young migrants and refugees placed in two refugee centers in Serbia in the time of COVID-19 pandemic

**DOI:** 10.1192/j.eurpsy.2023.142

**Published:** 2023-07-19

**Authors:** J. Vasic, Roberto Grujicic, Oliver Toskovic, Milica Pejovic Milovancevic

**Affiliations:** Institute of Mental Health, Belgrade, Serbia

## Abstract

**Abstract: Introduction:**

As the global resettlement needs are further increasing, the questions on refugee youths’ wellbeing arise. The experience of migration during childhood might interfere with the developmental trajectories in different ways. Refugee youths might be at higher risk of violence, abuse and mental health problems.

**Objectives:**

This study aimed to explore the prevalence of alcohol and substance use among young refugees, along with the indicators of experienced psychological difficulties.

**Methods:**

Data collection was followed by numerous difficulties–C-19 pandemic, linguistic diversity and high respondents’ illiteracy rate.

**Results:**

The sample consisted of 184 participants aged 11–18 years. More than a half of them displayed symptoms of PTSD - more frequently females, those who resided in a greater number of refugee centers and those who were exposed to abuse and domestic violence. Half of the respondents consumed energy drinks, slightly less than a third of them used tobacco, 13% consumed alcohol, 4.6% marijuana, whereby the frequency of other substance use was significantly lower. The significant indicators of individual propensity to use alcohol and substances were shown to be older age (14-18 years), male gender, lower education, being unaccompanied child and exposure to emotional abuse.

**Conclusions:**

Our research confirmed that young refugees, especially unaccompanied, might be at higher risk for mental health difficulties. Research on this topic should aim to link scientific data to sustainable practices, applicable in everyday life.

**Disclosure of Interest:**

None Declared

